# Can Phonotherapy Serve as an Adjunct Treatment for Acute and Chronic Stroke? A Preliminary Report

**DOI:** 10.3390/healthcare14121689

**Published:** 2026-06-12

**Authors:** Wiktor Rybicki, Katarzyna Kapcia, Marek Krzystanek, Anna Brzęk, Kamil Barański, Iwona Schuster, Dorota Szydlak, Wiktoria Balcerzak, Anetta Lasek-Bal

**Affiliations:** 1 Department of Neurology, Comprehensive Stroke Center, Upper-Silesian Medical Centre, Medical University of Silesia, 40-055 Katowice, Poland; wmrybicki@gmail.com (W.R.); ka.kapcia@gmail.com (K.K.); balcerzak.wiktoria97@gmail.com (W.B.); abal@sum.edu.pl (A.L.-B.); 2 Department of Clinical and Community Psychiatry and Psychology, Collegium Medicum, WSB University, 41-300 Dąbrowa Górnicza, Poland; krzystanekmarek@gmail.com; 3 Department of Physiotherapy, Faculty of Health Sciences in Katowice, Medical University of Silesia, 40-055 Katowice, Poland; abrzek@sum.edu.pl; 4 Department of Epidemiology, Faculty of Medical Sciences in Katowice, Medical University of Silesia, 40-055 Katowice, Poland; kbaranski@sum.edu.pl; 5 Psychiatric Rehabilitation Ward, Upper-Silesian Medical Centre, Medical University of Silesia, 40-055 Katowice, Poland; iwonaschuster@gmail.com; 6 Department of Neurology, School of Health Sciences, Medical University of Silesia, 40-055 Katowice, Poland

**Keywords:** stroke, phonotherapy, mRS, MoCA

## Abstract

Stroke is a leading cause of morbidity and long-term disability worldwide. This study evaluated the feasibility, safety, and preliminary clinical effects of phonotherapy (PHT) as an adjunct to standard care in patients with acute ischemic stroke. This prospective observational study enrolled 140 patients, who were assigned to receive either phonotherapy in addition to standard care (PHT group, *n* = 70) or standard care alone (control group, *n* = 70). Phonotherapy consisted of twice-daily 528 Hz sound stimulation administered for 3 months. Neurological (NIHSS), functional (mRS), and cognitive (MoCA) outcomes were assessed at days 10 and 90. At day 10, patients receiving PHT showed significantly better neurological, functional, and cognitive outcomes compared to the controls. However, these differences were not sustained at 90 days. Phonotherapy was not an independent predictor of favorable functional outcome at 90 days. Recurrent stroke occurred in three patients (4.3%) in the PHT group and nine (12.9%) in the control group (*p* = 0.07). No intervention-related adverse events were observed. Phonotherapy appears to be a safe adjunct intervention in acute ischemic stroke and may be associated with short-term improvements in selected outcomes. Overall, phonotherapy appeared safe as an adjunctive intervention in patients with acute ischemic stroke and showed possible short-term associations with improvements in selected outcomes, although these preliminary findings require confirmation in randomized controlled trials.

## 1. Introduction

Stroke remains a leading cause of morbidity, long-term disability, and mortality worldwide [1,2,3,4]. Evidence-based acute management includes reperfusion therapies and early rehabilitation aimed at improving functional and cognitive outcomes [5,6,7,8]. Current clinical practice follows established guidelines [9,10], while preventive strategies aim to reduce recurrence risk [11,12].

Music- and sound-based interventions have been explored as supportive approaches in neurological rehabilitation, primarily targeting cognitive, emotional, and motor outcomes [13,14,15]. However, their potential direct neurobiological effects remain insufficiently understood. Music-based rehabilitative interventions include rhythmic auditory stimulation to support movement, as well as music listening and singing to improve mood, promote well-being, and reduce pain [16,17,18]. In animal models, exposure to music has been shown to stimulate synaptic plasticity, neurotrophin production, and neurogenesis, while also supporting cognitive-motor recovery [19]. In stroke patients, music- and sound-based therapies appear to offer a promising means of increasing physical, social, and cognitive activity [20].

Current evidence suggests that music therapy may improve cognitive and motor function and accelerate recovery [21]. Studies suggest that both MBIs and musical training engage multiple cortical and subcortical networks, encompassing perceptual, sensorimotor, cognitive, and emotional processes [22]. Current evidence indicates that listening to sounds and music may induce structural white matter neuroplasticity in patients with left hemispheric brain damage, while preserved musical abilities in the right hemisphere may support compensatory recovery. Melody-based treatment has also been shown to improve connectivity between motor speech control regions, including the bilateral supplementary motor areas and insulae, and right hemispheric language areas, such as the inferior frontal gyrus [23].

Phonotherapy (PHT) is based on structured sound stimulation at specific frequencies, acting through auditory and potentially multisensory pathways.

It utilizes selected, musical frequencies, unlike music therapy, which uses musical pieces or melodies with varying ranges of sounds (i.e., broad and unspecified range of sound frequencies). Using individual sounds allows for the identification of frequencies that can have a health-promoting effect on the human body.

Experimental and preliminary clinical studies suggest that sound-based stimulation may influence neuroplasticity, cerebral perfusion, and cognitive processes [15,24,25]. However, clinical evidence in stroke populations remains limited [26,27,28,29].

In view of the lack of randomized controlled trials, this observational study was designed to assess the feasibility, safety, and preliminary effects of PHT as an adjunct to standard stroke care.

## 2. Materials and Methods

### 2.1. Participants

Patients hospitalized with acute ischemic stroke between September 2022 and December 2023 were considered eligible for this prospective observational study. Inclusion criteria were first-ever ischemic stroke and a baseline neurological deficit ≤ 15 points on the National Institutes of Health Stroke Scale (NIHSS) on day 1. Patients with hemorrhagic transformation or transient ischemic attack were excluded.

A total of 158 patients were initially enrolled; 140 patients completed the study (70 in the PHT, 70 in the control). A power analysis indicated that, at a significance level of α = 0.05, the sample size provided 80% power to detect an effect size of Cohen’s d = 0.47, corresponding to a moderate between-group difference.

**Figure 1:**
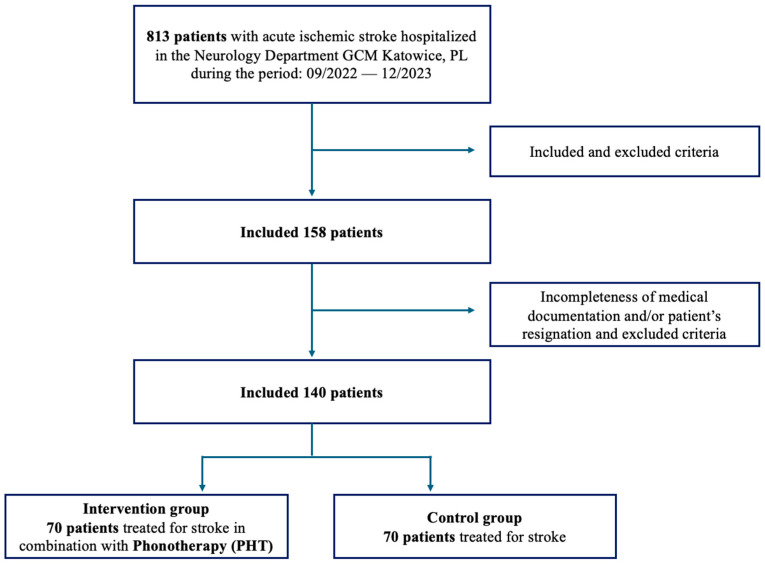
Flow diagram of patient selection, allocation, and final study cohort (*n* = 140).

The participant selection process is presented in Figure 1.

### 2.2. Study Design and Outcomes

In this observational study, patients received either PHT in addition to standard care or standard care only. Group assignment followed an alternating sequence, with every second patient allocated to the PHT plus standard care group. Baseline variables included age, sex, vascular risk factors, OCSP stroke subtype, and ASCOD classification.

Neurological status was assessed using NIHSS (days 1 and 10), functional outcome using mRS (days 10 and 90), and cognitive status using the MoCA (days 10 and 90). Major adverse cardiovascular events were recorded during the 90-day follow-up.

### 2.3. Intervention

Patients received a phone equipped with headphones and an application containing a set of sessions with sounds. PHT consisted of 528 Hz sound stimulation embedded in a musical background delivered via a mobile application (lifeAPP, Science2B, Chorzów, Poland), using the earphones.

PHT is a contemporary form of a music-based therapy that uses selected musical frequencies rather than specific musical compositions, allowing the intervention to be quantified and evaluated methodologically. The choice of low-frequency sounds was informed by a review of the scientific literature on the clinical effects of sound frequencies on the human body [15]. Specific frequencies were selected with reference to Pythagorean tuning, a musical tuning system in which interval frequency ratios are determined by choosing a sequence of fifths [30]. Following consultation with a music composer, consonant frequencies perceived as harmonious to the human ear were selected.

Therapy started on day 3 post-stroke. Patients received two 30 min sessions daily for 3 months. Patients were advised to undergo PHT in a quiet room in the afternoon. The study coordinator maintained telephone contact with patients at least once per week to monitor adherence, address potential problems, and support PHT administration. The control group received standard care only. The study team consisted of neurologists, including two physicians responsible for patient qualification and four examining physicians, as well as three psychologists. Apart from the qualifying physicians, all of the remaining team members were blinded to group assignment.

### 2.4. Statistical Analysis

Continuous variables were analyzed using a *t*-test or Wilcoxon test, and categorical variables using a chi-square or Fisher’s test. Logistic regression was used to identify predictors of favorable outcome (mRS ≤ 2). Due to sample size limitations, multivariate adjustment was limited. Statistical significance was set at *p* < 0.05.

### 2.5. Ethics Statement

The study was approved by the Bioethics Committee of the Medical University of Silesia and conducted in accordance with the Declaration of Helsinki. Written informed consent was obtained from all of the participants.

## 3. Results

A total of 140 patients were included.

The median age was 63 years (IQR 16), with no difference between the groups. Baseline characteristics were comparable except for higher prevalence of diabetes mellitus and obesity in the PHT group. Baseline demographic and clinical characteristics are presented in Table 1.

Baseline NIHSS did not differ between the groups (5.5 ± 5.4 vs. 5.8 ± 5.3; *p* = 0.3). At day 10, NIHSS was significantly lower in the PHT group (2.1 ± 2.5 vs. 3.3 ± 3.3; *p* = 0.02).

**Figure 2:**
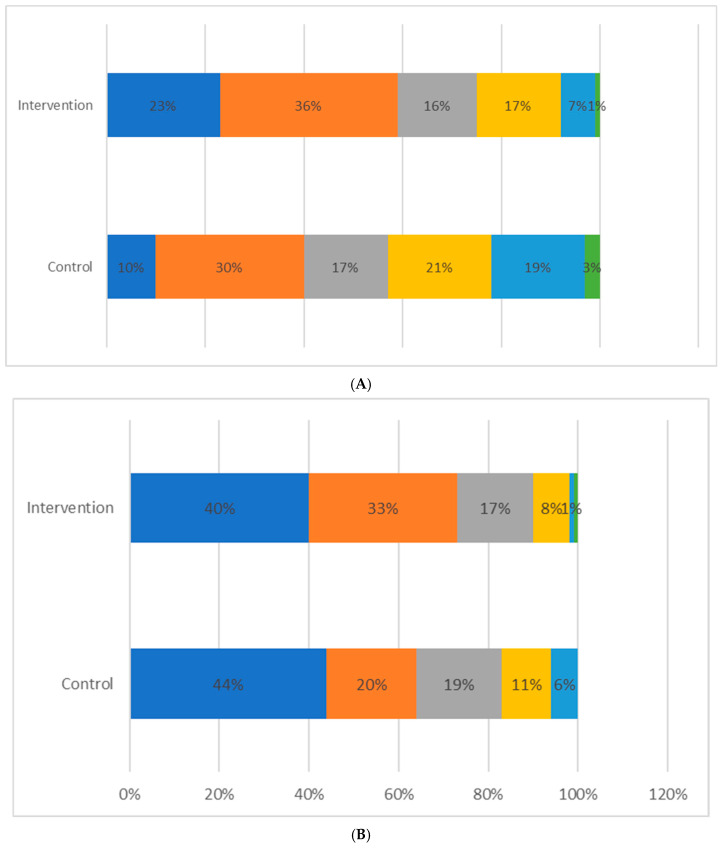
Distribution of the modified Rankin Scale (mRS) scores in the intervention and control groups at day 10 (**A**) and day 90 after stroke onset (**B**). mRS 0—navy blue, mRS 1—orange, mRS 2—grey, mRS 3—yellow, mRS 4—blue, mRS 5—green.

Functional outcomes assessed via the mRS showed a significant difference at day 10 in favor of the intervention group (*p* = 0.007), while no significant difference was observed at day 90. The distribution of mRS scores is presented in Figure 2A,B.

Cognitive outcomes assessed using the MoCA were significantly higher in the intervention group at day 10 (24 ± 4.7 vs. 20.5 ± 7.4; *p* = 0.004), while no differences were observed at day 90 (23.5 ± 6.2 vs. 24.5 ± 4.6; *p* = 0.9).

In univariate analysis, age, baseline NIHSS, NIHSS at day 10, and the MoCA at day 10 were associated with favorable functional outcome (mRS ≤ 2 at day 90). In multivariate analysis, baseline NIHSS, diabetes mellitus, and the MoCA at day 10 remained significant predictors, while phonotherapy was not an independent predictor. Results of multivariate logistic regression are presented in Table 2.

During the 90-day follow-up, recurrent stroke occurred in three patients (4.3%) in the intervention group and nine patients (12.9%) in the control group (*p* = 0.07). No other major adverse cardiovascular events or phonotherapy-related adverse effects were observed.

## 4. Discussion

Phonotherapy is a novel and insufficiently explored therapeutic approach proposed as a supportive intervention in patients with neurological disorders [15]. The primary aim of this study was to evaluate its feasibility, safety, and preliminary clinical effects in patients with acute ischemic stroke.

The results of this prospective non-randomized study suggest that PHT may be associated with short-term improvement in neurological and functional outcomes. At day 10, patients receiving PHT demonstrated significantly better neurological status (NIHSS) and functional performance (mRS), as well as higher cognitive scores (MoCA), compared to the controls. However, these differences were not sustained at 90-day follow-up, where no significant between-group differences were observed.

Importantly, multivariate analysis did not confirm phonotherapy as an independent predictor of favorable long-term functional outcome. These findings indicate that the observed early benefits may be influenced by baseline clinical characteristics, recovery dynamics, or non-specific effects related to increased stimulation during early rehabilitation.

Previous studies on music- and sound-based interventions suggest potential benefits in neurorehabilitation, particularly in cognitive and mood-related outcomes [13,14,15,20,21,22]. However, most available evidence concerns heterogeneous neurological populations and differs substantially in intervention type, intensity, and methodological quality. Therefore, direct comparison with phonotherapy is limited.

The biological mechanisms potentially underlying sound-based interventions remain speculative. In the rehabilitation context, music may be regarded as a form of environmental enrichment that promotes activity-dependent neuroplasticity within the large-scale brain networks [31]. Experimental data suggest that auditory and vibrational stimulation may influence neuroplasticity, cerebral perfusion, and neurochemical pathways, including dopaminergic and inflammatory systems [24,25,31,32]. PHT, like other sound-based therapies, may act on brain regions involved in processing tonal and rhythmic information.

Emotional and cognitive responses to auditory stimuli and music are well documented, supporting their relevance in brain modulation [33,34,35,36,37,38,39]. The emotional component of music activates several brain regions involved in emotional processing, including the insular and cingulate cortices, hippocampus, amygdala, hypothalamus, and prefrontal cortex [40,41]. The long-term efficacy of sound and music therapy was examined in a three-arm randomized controlled trial comparing daily music listening with a control intervention, audiobook listening, and standard care in patients who had experienced stroke. Music listening improved recovery of verbal memory and attention and was associated with a reduced depressive mood [42]. Another study also showed that exposure to music increased gray matter volume in spared prefrontal and limbic areas in patients with left hemisphere lesions [43]. A recent study reported that daily music listening, either alone or in combination with mindfulness training, improved verbal memory and attention more than audiobook listening [44]. Although these finding suggest that music listening may have cognitive, emotional, and neural benefits after stroke, its more tailored use in stroke rehabilitation requires a clearer understanding of which musical components drive these effects and which patients are most likely to benefit.

From a rehabilitation perspective, early post-stroke recovery is strongly influenced by multimodal stimulation, including physical therapy, cognitive engagement, and environmental enrichment. Structured neurorehabilitation approaches have been shown to support functional recovery and neuroplasticity after stroke [45,46,47,48,49]. An individualized approach to post-stroke rehabilitation, including music-based therapy, may therefore be particularly useful. In this context, phonotherapy may act as an additional sensory stimulation modality; however, its additive value over standard rehabilitation remains uncertain.

Given the exploratory nature of this study, the findings should be interpreted cautiously.

The absence of sustained long-term effects and lack of independent predictive value of PHT highlight the need for randomized controlled trials with larger sample sizes and standardized protocols.

### Limitations

We identified several limitations of this study, including its single-center design, non-randomized allocation, potential baseline imbalances and relatively small sample size. Differences in the prevalence of diabetes and arterial hypertension between groups may have influenced stroke outcomes.

Despite these limitations, the study findings are relevant from both scientific and clinical perspectives. The study was conducted in a relatively homogeneous patient population and used validated diagnostic tools and objective outcome measures, which supports the reliability of the results. Therefore, the findings may have practical implications for clinical practice.

## 5. Conclusions

Phonotherapy may be a safe adjunctive intervention in patients with acute ischemic stroke and may be associated with early improvements in neurological and functional outcomes. However, these findings were not sustained at 90 days, and PHT was not an independent predictor of long-term outcome. Further randomized controlled studies are required to confirm these preliminary results and to define the role of PHT in stroke rehabilitation.

## Figures and Tables

**Table 1:** Baseline clinical and demographic characteristics of the study population.

Variable		Group		*p* Value *
		Intervention	Control	
Age [years; X ± SD]		64 ± 17	61.5 ± 14	0.6
Sex	M; %	43; 61.4%	46; 65.7%	0.5
	F; %	27; 38.6%	24; 34.3%	
Stroke type (OCSP)	PACI	15; 21.4%	20; 28.6%	0.2
	LACI	23; 32.8%	29; 41.4%	
	TACI	22; 31.4%	15; 21.4%	
	POCI	10; 14.2%	6; 8.6%	
Stroke phenotype (ASCOD)	C	22; 31.4%	24; 34.3%	0.09
	S	18; 25.7%	22; 31.4%	
	A	25; 35.7%	17; 24.3%	
	D	3; 4.3%	0	
	O	2; 2.9%	7; 10%	
Treatment of ultra-acute stroke	Reperfusion therapy	30; 42.9%	32; 46.3%	0.6
	Other	40; 57.1%	37; 53.7%	
Arterial hypertension		52; 74.3%	56; 80%	0.4
Diabetes mellitus		18; 25.7%	37; 52.9%	0.001
Atrial fibrillation		11; 15.2%	8; 11.6%	0.4
Smoking		24; 34.3%	26; 37.1%	0.7
Myocardial infract up to 30 days		0	3; 4.3%	0.2
Carotid stenosis ≥ 60%		11; 15.7%	8; 11.4%	0.4
Obesity		5; 7.1%	20; 28.6%	<0.001
Dyslipidemia		47; 67.1%	46; 65.7%	0.8

* probability value. Abbreviations: M—male, F—female, OCSP—Oxfordshire Community Stroke Project, PACI—partial anterior circulation infarct, LACI—lacunar infarct, TACI—total anterior circulation infarct, POCI—posterior circulation infarct, ASCOD phenotyping (A: atherosclerosis; S: small-vessel disease; C: cardiac pathology; O: other causes; D: dissection).

**Table 2:** Factors associated with favorable functional outcome (mRS ≤ 2) at day 90: multivariate logistic regression analysis.

Dependent Variable	Variable	OR 95% CI
mRS ≤ 2 on day 90	Age	0.95 (0.91–1.00)
	Sex (Female ref.)	1.31 (0.49–3.52)
	Reperfusion vs. other	N/A
	Arterial hypertension	0.35 (0.07–1.63)
	Diabetes mellitus	1.47 (0.52–4.14)
	Smoking	1.65 (0.56–4.91)
	MoCA on day 10	1.09 (1.02–1.16)
	Carotid stenosis	0.53 (0.15–1.81)
	CHD/MI	0.47 (0.18–1.26)
	Obesity	0.78 (0.23–2.61)
	Dyslipidemia	1.18 (0.43–3.23)
	Recurrent stroke	0.42 (0.10–1.75)
	NIHSS on day 1	0.91 (0.84–0.98)
	NIHSS on day 10	0.67 (0.56–0.80)
	Phonotherapy	0.53 (0.19–1.45)

Abbreviations: MoCA—Montreal Cognitive Assessment; CHD/MI—Coronary Heart Disease/Myocardial Infarction; NIHSS—National Institutes of Health Stroke Scale. Notes: OR—odds ratio; CI—confidence interval.

## Data Availability

The raw data supporting the conclusions of this article will be made available by the authors on request.
